# Clinical evaluation of day surgery for anal fistula excision with MRI-assisted diagnosis: a retrospective analysis of 121 cases

**DOI:** 10.3389/fmed.2025.1579652

**Published:** 2025-05-27

**Authors:** Shaohua Zhang, Fangying Chen, Yifan Wei, Xiaolu Ma, Haidi Lu, Youyu Luo, Liqiang Hao, Jianping Lu, Yonggang Hong

**Affiliations:** ^1^Department of Colorectal Surgery, Changhai Hospital, Second Military Medical University, Shanghai, China; ^2^Department of Radiology, Changhai Hospital, Second Military Medical University, Shanghai, China

**Keywords:** anal fistula, surgical treatment, day surgery, clinical practice, MRI

## Abstract

**Objective:**

The clinical practice of anal fistula excision surgeries conducted in the day surgery unit was analyzed, and the efficacy and safety of this surgery were evaluated.

**Methods:**

The clinical data of 121 patients with anal fistulas who underwent excision surgery at Changhai Hospital from October 2021 to April 2024 was retrospectively analyzed, including age, gender, body mass index (BMI), diagnosis, fistula characteristics (number and locations of internal and external openings), preoperative magnetic resonance imaging (MRI) of fistulas, surgical and anesthetic methods, length of hospital stay, verbal rating scale (VRS), outpatient follow-up frequency, wound healing status, rehabilitation time, postoperative complications, and patient satisfaction. Kappa statistics were employed to evaluate the value of MRI in assessing the Parks classification and the number of fistulas compared with that of intraoperative findings.

**Results:**

All 121 patients successfully underwent anal fistula excision or excision combined with seton placement, with no patients lost to follow-up. All patients fully recovered, achieving a cure rate of 100%. Postoperative complications were rare, with only one patient (0.3%) experiencing pruritus and no recurrence. The rate of patient satisfaction reached 96.7%. The accuracies of the Park classification and number of fistulas assessed by MRI were 95.9 and 94.2%, respectively. The Kappa values were 0.948 and 0.848, respectively.

**Conclusion:**

Day surgery for anal fistula excision resulted in shorter hospital stays, lower medical costs, and fewer postoperative complications. Furthermore, MRI provided a reliable preoperative assessment of the Parks classification and the number of fistulas. Such an approach is a safe and feasible surgical treatment that deserves wider adoption.

## Highlights

- Compared with nonday surgery, day surgery is associated with shorter hospitalization times and lower costs.
- Patients with anal fistula who underwent day surgery experienced fewer postoperative complications and had a zero long-term recurrence rate.
- Preoperative MRI of fistulas can be used to assess both the Parks classification and the number of fistulas effectively.

## Introduction

Anal fistula, a prevalent condition in colorectal surgery, is characterized by a granulomatous tract connecting the anal canal or rectum to the perianal skin and generally consists of an internal opening, a fistula tract, and an external opening. The disease is commonly observed in young and middle-aged men, typically between the ages of 20 and 40. The clinical assessment of anal fistula begins with digital rectal examination, which initially involves visual inspection to document the number, location, and discharge characteristics (e.g., purulent, serosanguinous) of external openings, followed by systematic palpation to map the fistula tract orientation, identify indurated areas, and evaluate tenderness. In select cases, cautious probing with a calibrated instrument may supplement physical examination to preliminarily trace the fistula course, though excessive manipulation is avoided to prevent iatrogenic false tract formation. Magnetic resonance imaging (MRI) serves as the gold standard for comprehensive preoperative evaluation, utilizing high-resolution T2-weighted sequences to delineate anatomical relationships between fistulous tracts and the sphincter complex, contrast-enhanced sequences to distinguish abscess cavities from fibrotic scarring, and diffusion-weighted imaging (DWI) to quantify active inflammation through restricted diffusion patterns (1). A retrospective study (2) demonstrated that the apparent diffusion coefficient (ADC) value of the fistula showed a significant negative correlation with MRI score (*p* = 0.002). In patients with anal fistula complicated by perianal abscess, the recurrence group exhibited significantly lower ADC values compared to the non-recurrence group (*p* = 0.03), whereas no statistically significant difference in ADC values was observed between recurrence and non-recurrence groups in simple anal fistula cases. Preoperative diffusion-weighted magnetic resonance imaging (DW-MRI) provides substantial prognostic value for anal fistula evaluation, with complex anal fistula (characterized by multiple fistulous tracts and prolonged disease duration) and modifiable lifestyle factors (including fatigue, consumption of spicy/greasy foods, and diarrhea) identified as primary risk factors for postoperative recurrence. For complex or recurrent cases, examination under anesthesia (EUA) remains indispensable, with its diagnostic accuracy significantly enhanced when correlated with MRI findings. Complementary laboratory investigations include inflammatory marker quantification (C-reactive protein, erythrocyte sedimentation rate) to assess systemic inflammatory burden, alongside pus cultures to exclude tuberculosis or Crohn’s disease-associated etiologies. This multimodal diagnostic framework, endorsed by contemporary guidelines, optimizes therapeutic planning while minimizing procedural risks.

Most anal fistulas result from infections in the anal crypts (anal glands), generating glandular fistulas that usually do not heal spontaneously. Early surgical intervention is required if there are no contraindications, including surgeries that damage the sphincter muscles (such as fistulotomy, seton placement, and fistulectomy) and surgeries that preserve sphincter function [such as transanal opening of intersphincteric space (TROPIS), ligation of intersphincteric fistula tract (LIFT), rectal mucosal advancement flap, fibrin glue closure, anal fistula plug (AFP), laser ablation, and video-assisted anal fistula treatment (VAAFT)]. Surgery can effectively treat fistulas in cases where the sphincter muscles are damaged, possibly accompanied by complications such as bleeding, infection, anal stricture, significant postoperative pain, potential sphincter damage, and even incontinence. Procedures that preserve the sphincter muscles avoid these complications, maintaining the anatomical integrity and normal function of the anus. However, these methods, such as mesenchymal stem cell transplantation (3, 4), local fistula injection of allogeneic human amnion epithelial cells (hAECs) (5), Darvadstrocel (6), autologous adipose-derived stem cells (ADSCs) (7, 8), and bone marrow-derived mesenchymal stromal cells (BMSCs) (9), which have relatively high cure rates, may not completely eliminate the fistula tract, leading to high recurrence rates and low long-term cure rates (10). The last three modalities are mainly used for the treatment of fistulas caused by Crohn’s disease and complex fistulas (11, 12). Anti-tumor necrosis factor (TNF) treatment is recommended for perianal fistulas in patients with Crohn’s disease. The consideration of simultaneous combined surgical closure in patients suitable for surgical treatment may also promote long-term MRI healing rates (13). Postoperative packing of the perianal abscess cavity (PPAC2) increases patient pain, and there is no significant difference in anal fistula formation or abscess recurrence with no packing. Some surgeons in China have attempted to improve the surgical approach to reduce the possibility of postoperative fistula formation, such as radical perianal abscess surgery or perianal abscess incision and drainage combined with one-stage hanging (14). All patients in this study underwent traditional fistulectomy or fistulectomy combined with seton placement (15). Fistulotomy generally offers advantages in terms of shorter surgical and healing times but a relatively higher recurrence rate. Conversely, fistulectomy allows for more thorough removal of pathological tissue, reducing the risk of recurrence. Therefore, surgical methods should be chosen on the basis of the patient’s condition.

Day surgery is primarily suitable for lower-risk, technically mature, and small-to-medium elective surgeries. The colorectal and anal day surgeries often include procedures for colorectal polyps, hemorrhoids, anal fistulas, anal fissures, rectal prolapse, pilonidal cysts, and sacral cysts. Despite its advantages, not all anal fistula patients are suitable for day surgery. Low and simple anal fistulas are relatively straightforward, with short postoperative dressing change cycles and a low incidence of complications such as bleeding, infection, and pseudohealing. Such fistula day surgeries are most suitable for the patient in good condition. Other types of anal fistulas require comprehensive assessment by a physician to determine their suitability for day surgery. In addition, several complications may occur after surgery (e.g., bleeding, infection, urinary retention, anal stenosis, anal incontinence, anal overflow, changes in anal morphology, and wound bridging or delayed healing).

Herein, the clinical practice of day surgery for anal fistula excision surgeries was investigated, and the efficacy and safety of this surgery were evaluated.

## Methods

### Study population

The clinical data and follow-up records of 121 patients who underwent day surgery for anal fistula excision at Changhai Hospital from October 2021 to April 2024 were retrospectively collected. This retrospective study received approval from the local ethics committee, and informed consent was waived because of its retrospective nature.

### Inclusion criteria

The inclusion criteria for the study were as follows: (1) confirmed diagnosis of anal fistula through imaging and postoperative pathological data, meeting the admission standards of the day surgery unit (age < 70 years, no severe comorbidities, good general condition, etc.); (2) complete postoperative follow-up data with a minimum follow-up period of 3 months; and (3) surgeries (either fistulectomy or fistulectomy combined with seton placement) performed by the same surgeon.

### Exclusion criteria

Patients were excluded if they had any of the following conditions: (1) anal fistulas associated with perianorectal abscess, perineal gangrene, anorectal stenosis, or inflammatory bowel disease. (2) A recent (within 3 months) history of sclerotherapy and anorectal surgery. (3) Presence of immunocompromised or coagulopathic disorders or the current need for anticoagulant therapy. (4) Pregnancy, persistent constipation, pelvic tumors, portal hypertension, Budd–Chiari syndrome, or inability to tolerate surgery.

### Classification of anal fistulas

There are multiple ways to classify anal fistulas, with the Parks classification being one of the most recognized types of fistulas (1, 16, 17). This classification categorizes fistulas based on the course of the fistula tract from the anal canal to the skin and their relationship to the internal and external sphincters: intersphincteric (the most common type, accounting for approximately 70% of cases), transsphincteric (representing approximately 25% of cases), suprasphincteric (occurring in approximately 4% of cases), extrasphincteric (the rarest type, comprising approximately 1% of cases), and superficial, playing an instrumental role in clinical diagnosis and treatment planning. Anal fistulas can also be divided into simple and complex types (18). Simple fistulas are typically found in initial cases, whereas complex fistulas are rare in primary glandular fistulas (approximately 5%) but more common in recurrent cases and those associated with Crohn’s disease, comprising 50–75% of all cases.

### Preoperative preparation

Patients experienced routine preoperative evaluations, including blood and urine tests, liver and kidney function assessments, blood glucose levels, coagulation function tests, electrocardiograms, chest X-rays, colonoscopy, abdominal ultrasound, and MRI of fistulas (19). Patients without significant abnormalities were arranged for surgery. Preoperative health education was provided to inform patients about surgical risks, perioperative precautions, and psychological care to alleviate tension. Patients were instructed to empty their bowels before the procedure, without routinely using oral laxatives. Assistance with defecation or enemas was provided in cases of constipation. Patients were advised to abstain from food and water after 10:00 p.m. before surgery. If necessary, functional drinks (e.g., sugar water) can be consumed before midnight.

### MRI of fistulas

Preoperative MRI of the fistulas was performed via an external phased-array coil, with all patients fasting for at least 4 h prior to imaging. The imaging protocol included sagittal T2-weighted imaging (T2WI) without fat suppression, axial-oblique high-resolution T2WI (short axis, without fat suppression), coronal-oblique high-resolution T2WI (long axis, without fat suppression), and gadolinium contrast-enhanced axial, sagittal, and coronal T1-weighted imaging (venous phase, with fat suppression). The Parks classification and the number of fistulas were independently determined by two radiologists (FY. C. and SY. M.). Any discrepancies between the two radiologists were resolved by an expert (F. S.) with over 15 years of experience in MRI radiology, who provided the final assessment.

### Surgical procedures

All patients in this study underwent fistulectomy combined with thread-drawing therapy. Surgical management was standardized according to Parks classification determined by preoperative MRI and intraoperative exploration findings (Intersphincteric/Transsphincteric fistulas: Retrograde anatomically precise fistulectomy with complete fistulous tract denudation. Suprasphincteric/Extrasphincteric fistulas: The above procedure combined with seton placement). The patients were placed in the prone position after anesthesia. The chief surgeon first examined the fistula to determine its course and depth, particularly focusing on complex or branched fistulas, the Parks classification, and the number of fistulas. The skin around the external opening or blind fistula was incised, and the entire fistula and branched fistulas were thoroughly removed or excised following the principles of anatomical resection in a retrograde manner. More importantly, the internal opening was identified and excised.

Ligation therapy was adopted for cases where the internal opening was too high or the fistula passed above the anorectal sphincter, which avoided incontinence caused by cutting the anorectal ring entirely at once. In detail, the subcutaneous and superficial portions of the external sphincter and the fistula below it were incised, and then a band was threaded from the remaining duct opening to the internal opening to bind the anorectal ring. Finally, the skin and subcutaneous tissue at the wound edges were excised to open the wound fully, which was then filled with iodoform gauze or oil gauze for appropriate drainage.

The key to surgery lay in accurately identifying the internal opening, followed by performing excision, incision, or tunneling to completely remove the fistula tract (the main duct, branches, and communicating tracts). It is crucial to properly handle the relationship between the sphincter and the fistula tract, as well as ensure smooth postoperative wound drainage. Otherwise, surgical failure or serious complications may occur.

### Postoperative care

After the operation, patients were transferred to the day surgery unit for routine anti-inflammatory, rehydration, analgesic, and other symptomatic supportive therapies. Patients were instructed to lie flat for 6 h without a pillow and fast for 24 h allowing for small sips of water. The gauze packing in the wound was removed 24 h postsurgery. Patients were discharged the following morning after a physician confirmed the absence of complications such as bloody stool, black stool, abdominal pain, or bloating.

Patients were advised to follow discharge instructions at home, including taking prescribed medications and performing warm water sitz baths. A follow-up visit was scheduled with the attending surgeon 1 week postsurgery to check the wound. Granulation tissue should grow from the base outward to prevent incision edges from adhering, which may impair healing. Excessive granulation tissue should be trimmed promptly, and the wound should be expanded and trimmed in cases of residual fistulas, infections, or inversion of the wound edges. After the rubber band from thread-drawing therapy naturally fell off, wound healing began from the base, with the portion inside the anal canal healing first. Care was taken to prevent premature surface skin adhesion.

### Statistical analysis

The data were processed, organized, and analyzed via SPSS 26.0 (Statistical Product and Service Solutions 26.0) and MedCalc 15.2.2 (MedCalc Software Ltd., Belgium). Categorical data are described as [n (%)], and continuous data were tested for normality via the Shapiro–Wilk test. Nonnormally distributed data were described via [M (*P*_25_, *P*_75_)]. Kappa statistics were calculated to assess the agreement between radiologists and intraoperative evaluations for Parks classification and the number of fistulas. *p* < 0.05 was considered to indicate statistical significance.

## Results

### Patient characteristics

A total of 121 consecutive patients were enrolled in the study, comprising 112 males (92.6%) and 9 females (7.4%), with ages ranging from 11--72 years and a mean age of 35.60 years. Detailed demographic and clinical information on the patients is provided in Table 1.

**Table 1:** Demographic and clinical information.

Item	Value	Stats
Gender, *n* (%)	Male	112 (92.6)
	Female	9 (7.4)
Age, *M* (*P*_25_, *P*_75_)		34 (27, 42)
BMI (kg/m^2^), *M* (*P*_25_, *P*_75_)		24.91 (23.37, 26.95)
Hospital stay duration (days)		2

**Figure 1:**
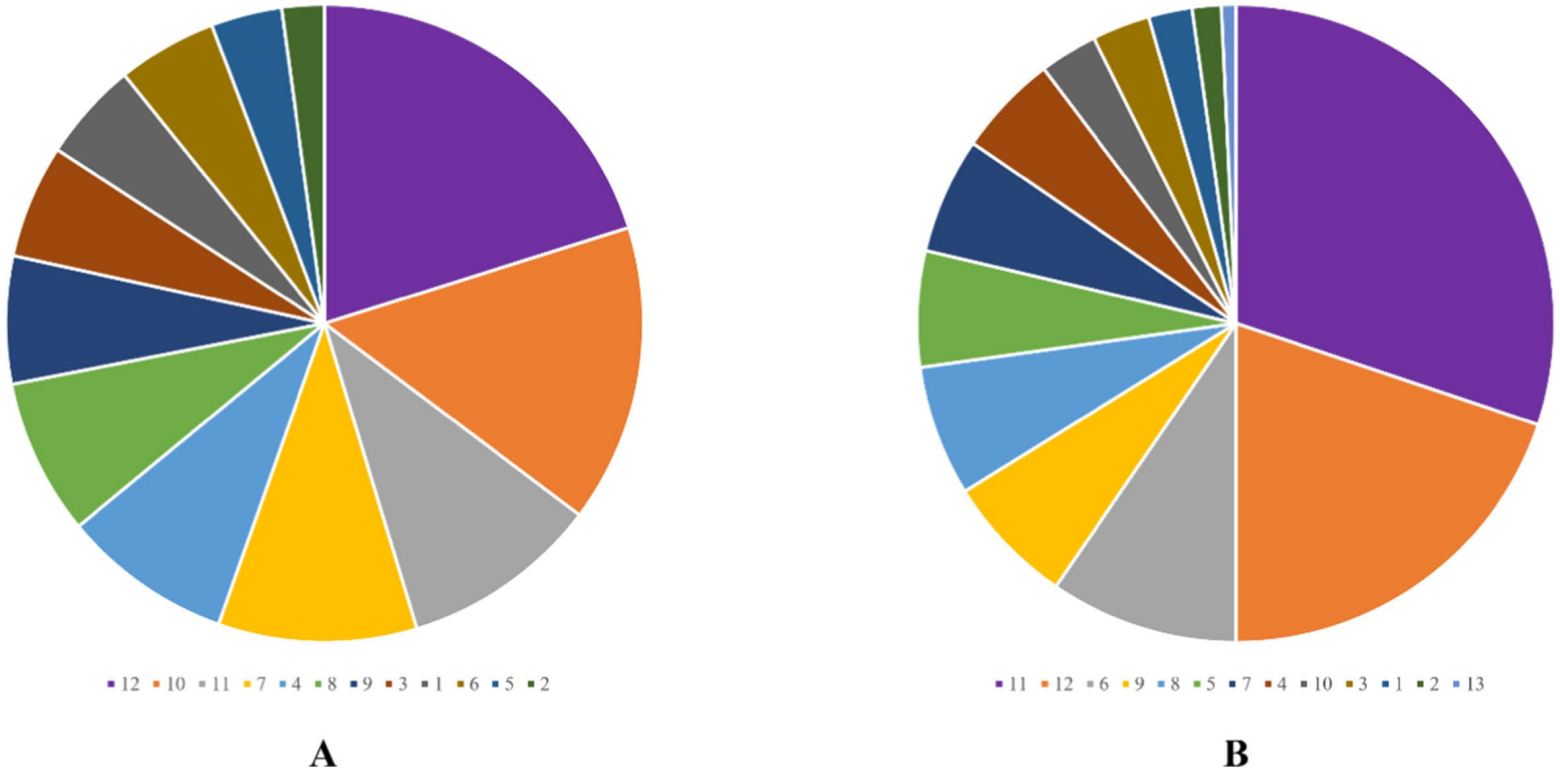
External opening scale **(A)** and internal opening scale **(B)**. The numbers represent the time direction at the Kraske-prone jackknife position.

Among the 121 patients, the majority of wounds healed well, with most patients having a single fistula (88.4%), nine patients (7.4%) presenting with two fistulas, and five patients (4.1%) comprising three fistulas. Postoperative bleeding was rare, with only three patients (2.5%) experiencing minor bleeding that did not require intervention. The detailed surgery-related indicators are summarized in Table 2. The external opening of the fistula was most commonly located at the 12 o’clock (28 patients, 20.1%) and 10 o’clock (21 patients, 15.1%) positions in the Kraske prone jackknife position (Figure 1A). For internal openings, the most frequent locations were at 11 o’clock (41 cases, 30.1%) and 12 o’clock (27 cases, 19.9%) in the same position (Figure 1B).

**Table 2:** Surgical indicators.

Indicator	Value	Stats, *n* (%)
Surgical method	Fistulectomy + thread-drawing therapy	6 (5.0)
	Fistulectomy	115 (95.0)
Number of fistulas**	1	107 (88.4)
	2	9 (7.4)
	3	5 (4.1)
Parks classification**	Intersphincteric	89 (73.5)
	Transsphincteric	25 (20.7)
	Suprasphincteric	6 (5.0)
	Extrasphincteric	0 (0)
	Superficial	1 (0.8)
Wound healing*	Poor	0 (0)
	Average	2 (1.6)
	Good	119 (98.3)
Postoperative bleeding	None	118 (97.5)
	Minor bleeding	3 (2.5)
	Requires intervention	0 (0)

*The healing situation of the wounds was determined by outpatient examination by the chief surgeon.

**Intraoperative findings.

### MRI assessment

**Figure 2:**
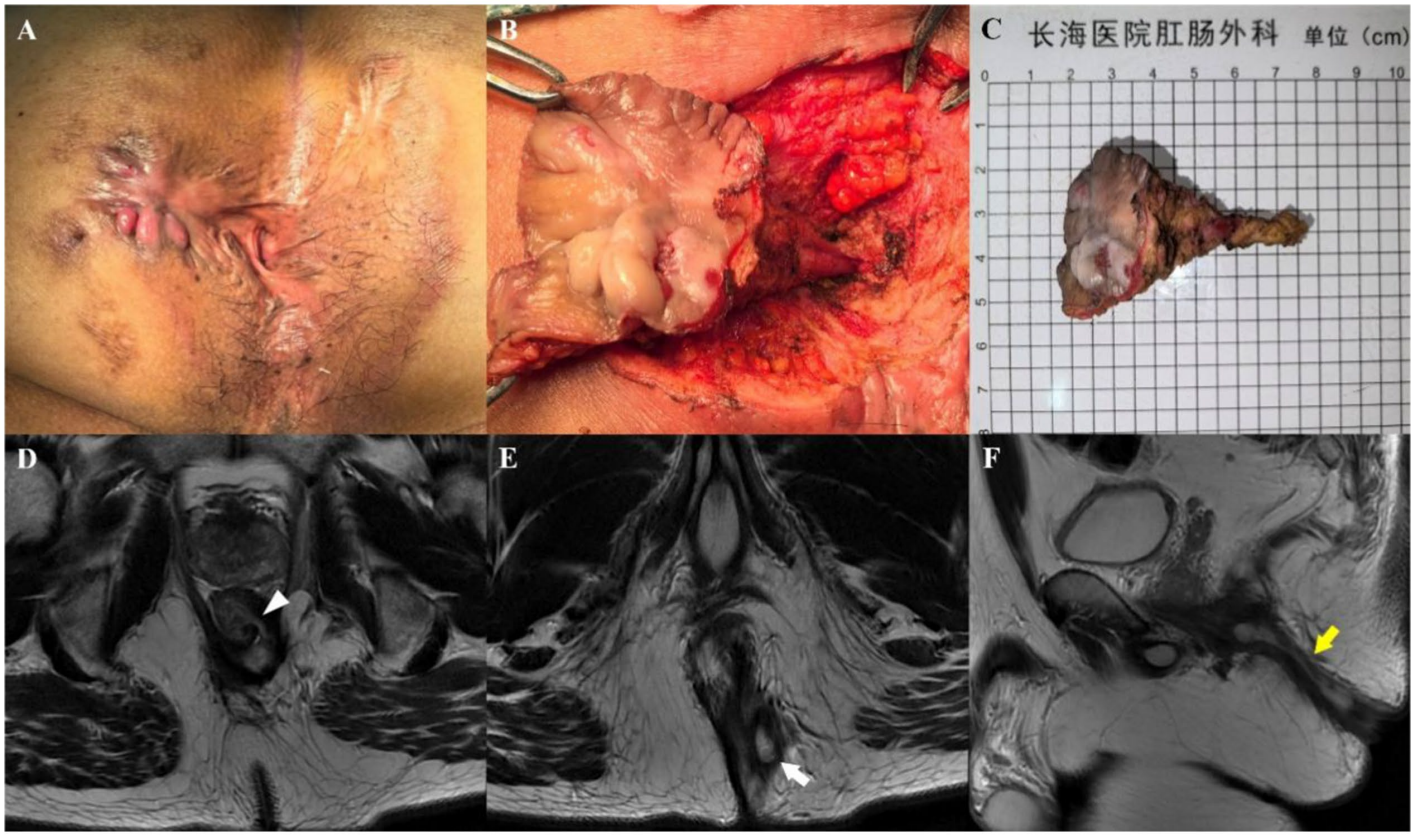
A 47-year-old man who underwent three anal fistula surgeries at another hospital. Preoperative appearance **(A)** and findings during the operation **(B)** showing a transsphincteric fistula. **(C)** Sample from surgical resection. Axial T2-weighted images without fat suppression show a left transsphincteric fistula crossing both layers of the sphincter complex, with an internal opening at the 3 o’clock position (**D**, arrowhead) and an external opening at the 5 o’clock position (**E**, arrow). Sagittal T2-weighted image without fat suppression **(F)** showing the transsphincteric extension of the fistula backward (yellow arrow). Preoperative MRI findings demonstrated complete concordance with intraoperative exploration.

**Figure 3:**
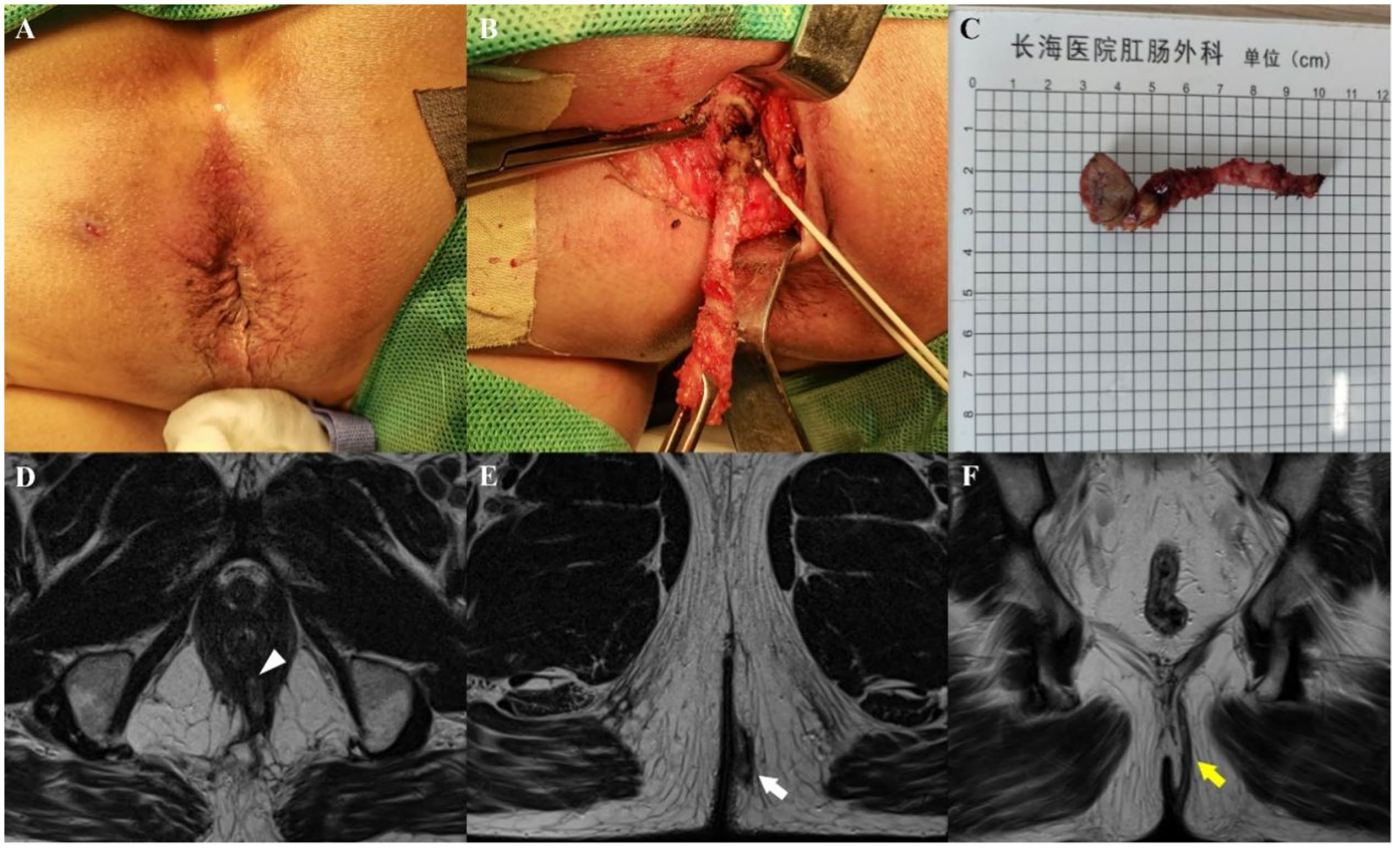
A 62-year-old man whose perianal purulent secretion had persisted for 6 months. Preoperative appearance **(A)** and findings during the operation **(B)** showing a suprasphincteric fistula. **(C)** Sample from surgical resection. Axial T2-weighted images without fat suppression show a left transsphincteric fistula crossing both layers of the sphincter complex, with an internal opening at the 6 o’clock position (**D**, arrowhead) and an external opening at the 5 o’clock position (**E**, arrow). Coronal T2-weighted image without fat suppression **(F)** showing upward fistula extensions through the intersphincteric space, over the top of the levator ani muscle, and then descending through the ischiorectal fossa at the left side to reach the skin (yellow arrow). Preoperative MRI findings demonstrated complete concordance with intraoperative exploration.

On preoperative MRI, the Parks classification was correctly diagnosed in 116 patients (95.9%, 116/121) according to the intraoperative findings. Similarly, the number of fistulas was accurately determined in 114 cases (94.2%, 114/121) from the intraoperative findings. The Kappa values for agreement between MRI and intraoperative findings were 0.948 (95% CI: 0.909–0.988) for the Parks classification and 0.848 (95% CI: 0.729–0.967) for the number of fistulas, as shown in Figures 2, 3.

### Postoperative recovery

Among the 121 patients, 108 (89.3%) attended outpatient follow-ups, and no cases of recurrence appeared. Postoperative pain was assessed to be mostly mild to moderate via the verbal rating scale (VRS), with 73 patients (60.3%) reporting mild pain and 27 patients (22.3%) experiencing moderate pain. Patient satisfaction was high, with 117 patients (96.7%) expressing satisfaction. The number of outpatient follow-up visits ranged from 2 to 5 times, and the median time required to resume normal activities was [21 (14, 30)] days. The detailed postoperative recovery indicators are listed in Table 3.

**Table 3:** Postoperative recovery indicators.

Indicator	Value	Stats, *n* (%)
Outpatient follow-up	No	13 (10.7)
	Yes	108 (89.3)
Recurrence	No	121 (100)
	Yes	0 (0)
Postoperative pain VRS score*	No pain	18 (14.9)
	Mild pain	73 (60.3)
	Moderate pain	27 (22.3)
	Severe pain	3 (2.5)
Patient satisfaction	Unsatisfied	4 (3.3)
	Satisfied	117 (96.7)

*Grade 0: No pain.

Grade I (mild pain): The patient feels pain but can tolerate it, is able to live normally, and sleep is not affected.

Grade II (moderate pain): The patient experiences significant pain that is unbearable and requires analgesics, with disturbed sleep.

Grade III (severe pain): The patient has severe pain that is unbearable, requires analgesics, has severely disturbed sleep, and may even be accompanied by autonomic system dysfunction or a positive position.

### Compliance

Most patients adhered to the prescribed postoperative care regimen. Celecoxib was taken as needed by 48 patients (39.7%), and diosmin was taken regularly by 115 patients (95.0%). Additionally, 118 patients (97.5%) performed regular warm sitz baths twice a day. Detailed compliance data are presented in Table 4.

**Table 4:** Descriptive statistics of patient compliance.

Indicator	Value	Stats, *n* (%)
Celecoxib usage	None	44 (36.4)
	Regular	29 (24.0)
	As needed	48 (39.7)
Diosmin usage	None	6 (5.0)
	Regular	115 (95.0)
	As needed	0 (0.0)
Warm sitz bath	None	3 (2.5)
	Regular twice daily	118 (97.5)
	Intermittent	0 (0.0)

### Complications

Postoperative complications were minimal. Among the 121 patients, only one case (0.8%) reported pruritus as a postoperative complication. Detailed data on complications are provided in Table 5.

**Table 5:** Complications.

Indicator	Value	Stats, *n* (%)
Anal stricture	No	121 (100.0)
	Yes	0 (0.0)
Perianal infection	No	121 (100.0)
	Yes	0 (0.0)
Anal incontinence	No	121 (100.0)
	Yes	0 (0.0)
Urinary retention	No	121 (100.0)
	Yes	0 (0.0)
Residual tissue prolapse	No	121 (100.0)
	Yes	0 (0.0)
Anal secretion	No	121 (100.0)
	Yes	0 (0.0)
Itching	No	120 (99.2)
	Yes	1 (0.8)

## Discussion

The technique of anal fistula pessary placement and early postoperative infection leading to pessary expulsion are common causes of failure. A multicenter randomized controlled trial reported that while anal fistula pessaries are costly, they do not significantly differ in quality of life for fecal incontinence or wound healing compared with other surgical modalities in the treatment of transsphincteric fistulas. The specific benefits and indications of pessary placement need further investigation (20). The timing of surgery is a crucial determinant of success. During the stable stage of an anal fistula, local inflammation diminishes, symptoms are alleviated, and the fistula tract becomes mature and well defined, which offers an optimal window for treatment options, including seton placement, excision, or fistulotomy. Operating during this stage facilitates the identification of the internal opening and enables complete excision of the fistula tract, resulting in a clean wound and favorable outcomes. Conversely, surgery at the wrong time results in a more complicated fistula, exacerbates the condition, increases the surgical difficulty, and potentially compromises the outcome.

Second, the choice of surgical method is a key factor. Various types of anal fistulas require tailored surgical approaches (21–23). There was no significant difference in postoperative complications or success rates between the drained mucosal flap technique and the rerouting Seton around the internal anal sphincter in the treatment of complex fistula-in-ano. The former has a longer healing time but a lower incidence of fecal incontinence (24). The surgical approaches should aim to achieve the best outcome while preserving anal function and minimizing surgical wounds to ensure the life quality of patients. The most critical aspect of anal fistula surgery is correctly identifying and addressing the internal opening. Complete excision or incision of the internal opening and the fistula tract is essential for successful surgery; otherwise, poor wound healing or recurrence may occur.

Currently, other centers recommend contains regular postoperative dressing changes to observe the wound promptly, the removal of foreign materials, pus, and necrotic tissue, appropriate drainage, and the reduced risk of irritation and infection. Inadequate dressing changes lead to poor wound healing or pseudohealing, and even recurrence. However, day surgery patients are not equipped with long-term inpatient care, and dressing changes in various medical settings or even at home increase the inconvenience and complexity of care and potentially compromise professionalism and expected outcomes. Due to the short hospital stay for day surgery patients, doctors cannot participate in postoperative dressing changes at our center. We recommend warm sitz baths and provide patients with written instructions and follow-up care.

All 121 patients were hospitalized for 2 days without delayed discharge, and the hospitalization time was significantly shorter than that in a traditional inpatient ward. All patients were cured, and 98.3% of the postoperative wounds healed well. Two patients experienced poor wound healing due to untimely postoperative dressing changes, and no recurrence occurred. There was only one case of itching among the postoperative complications, which may be caused by a large surgical wound with more exudation, as well as a change in anal morphology that led to exudate accumulation and irritation of the surrounding skin. The patient was instructed to take a warm water sitz bath after defecation and maintain cleanliness and dryness to relieve symptoms. Patient satisfaction was 96.7%, with 4 patients being dissatisfied because of a short hospital stay, even experiencing internal anxiety. They were discharged as out-of-town patients with painful wounds, long-distance transportation was deeply inconvenienced for them, and daily postoperative checkups and wound observations by the attending surgeon cannot be accepted.

Several key findings were revealed. First, it is crucial for us to strictly control the surgical admission timing. We recommend performing surgeries during the stable stage of the fistula when the tract is clear, the internal opening is evident, and no signs of infection are found. Preoperative determination of fistula phase (stable vs. active) was conducted through symptom duration assessment, digital rectal examination of fistula tract characteristics, and high-resolution contrast-enhanced rectal MRI. The complexity of the fistula is not a criterion for exclusion or admission to our day surgery unit. Second, reducing postoperative complications and recurrence rates is essential. Causes such as inflammatory bowel disease, tuberculosis, syphilis, and HIV must be excluded for patients with complex fistulas involving multiple external or internal openings, which are strictly controlled through preoperative examinations. The key factor affecting the incidence of complications and recurrence in the day surgery unit is the surgical method. Our center primarily adopts fistulectomy or fistulectomy combined with seton placement. Other surgical methods with strict postoperative follow-up and dressing changes, are not feasible in a day surgery setting. During surgery, we strictly control bleeding and recommend suturing the rectal mucosa at the internal opening after complete excision of the fistula tract to prevent postoperative bleeding during sitz baths and create a wide-mouthed and narrow-bottomed wound to prevent pseudohealing. A high-quality systematic evaluation revealed a high recurrence rate of high trans-sphincteric (25), undefined internal opening, and horseshoe-shaped extension of the anal fistula. Therefore, our center uses retrograde fistula resection to remove the fistula with a complete internal opening and allows the wound to become a wide-mouthed and narrow-bottomed pattern, which facilitates drainage and healing of the wound. Unlike other centers where strict dressing changes are required postoperatively, day surgery patients at our center are advised to perform warm sitz baths at approximately 40°C, 2–3 times a day for 15–20 min each. This method minimizes the likelihood of complications even if outpatient follow-ups are challenging. Despite these successes, our center still faces challenges such as managing patient anxiety before, during, and after surgery. It is crucial for our team to provide more information on patient counseling, explanations, etc., to address these psychological issues.

There are several limitations in this study. First, as a single-center retrospective study with a small number of patients, our findings need to be further validated by multicenter prospective studies. Second, when performing day surgery for anal fistula, surgeons are required to have the experience and skills to achieve a safe and successful operation, and strict discharge criteria (e.g., stable vital signs, no short-term serious complications) must be combined based on a short postoperative hospital observation time for patients. Additionally, day surgery for anal fistula requires hospitals to provide sufficient medical resources, a well-established management system (including anesthesia and nursing teams), and an independent and mature day surgery unit. Finally, strict patient selection criteria must be followed, considering factors such as age, basic conditions, comorbidities, communication abilities, and follow-up conditions to ensure surgical quality and patient safety. Adhering to these principles can maximize the benefits of day surgery, accelerating bed turnover, balancing medical resources, and achieving the goal of rapid recovery. In conclusion, day surgery for anal fistula is demonstrated to be feasible. This novel surgical treatment model could be a possible solution to save medical resources and seek medical opportunities in central cities.

This study retrospectively analyzed 121 cases of day surgery for anal fistula, confirming the significant clinical value of performing anal fistula excision in day surgery in a tertiary hospital setting. The results demonstrated a 100% cure rate (121/121) and an exceptionally low postoperative complication rate (0.3%), confirming the safety and efficacy of this surgical approach. With a 96.7% patient satisfaction rate, the study indicates that the day surgery model provides a positive healthcare experience for patients and optimizes the allocation of medical resources. This research provides evidence for the promotion of day surgery for anal fistula, offering valuable practical implications for improving healthcare efficiency and reducing medical costs. It is recommended that this approach be implemented in healthcare institutions that are equipped to meet the necessary conditions.

## Data Availability

The data analyzed in this study is subject to the following licenses/restrictions: the datasets generated or analyzed during the study are available from the corresponding author on reasonable request. Requests to access these datasets should be directed to 1401312182@qq.com.

## References

[1] Gaertner WB Burgess PL Davids JS Lightner AL Shogan BD Sun MY. The American Society of Colon and Rectal Surgeons clinical practice guidelines for the Management of Anorectal Abscess, fistula-in-Ano, and rectovaginal fistula. Dis Colon Rectum. (2022) 65:964–85. doi: 10.1097/DCR.0000000000002473, PMID: 35732009

[2] Liu X Wang Z Ren H Ren A Wang W Yang X. Evaluating postoperative anal fistula prognosis by diffusion-weighted MRI. Eur J Radiol. (2020) 132:109294. doi: 10.1016/j.ejrad.2020.109294, PMID: 33038577

[3] Wang H. Mesenchymal stem cells transplantation for perianal fistulas: A systematic review and Meta-analysis of clinical trials. Stem Cell Res Ther. (2023) 14:103. doi: 10.1186/s13287-023-03331-6 37101285 PMC10134595

[4] Wei J. Efficacy and safety of allogeneic umbilical cord-derived mesenchymal stem cells for the treatment of complex perianal fistula in Crohn’s disease: A pilot study. Stem Cell Res Ther. (2023) 14:311. doi: 10.1186/s13287-023-03531-0, PMID: 37904247 PMC10617053

[5] Keung C Nguyen TC Lim R Gerstenmaier A Sievert W Moore GT. Local fistula injection of allogeneic human amnion epithelial cells is safe and well tolerated in patients with refractory complex perianal Crohn’s disease: a phase I open label study with long-term follow up. EBioMedicine. (2023) 98:104879. doi: 10.1016/j.ebiom.2023.104879, PMID: 38042747 PMC10755113

[6] Furukawa S Mizushima T Nakaya R Shibata M Yamaguchi T Watanabe K. Darvadstrocel for complex perianal fistulas in Japanese adults with Crohn’s disease: a phase 3 study. J Crohns Colitis. (2023) 17:369–78. doi: 10.1093/ecco-jcc/jjac144, PMID: 36149832 PMC10069615

[7] Zhou C Li M Zhang Y Ni M Wang Y Xu D. Autologous adipose-derived stem cells for the treatment of Crohn’s fistula-in-Ano: An open-label, controlled trial. Stem Cell Res Ther. (2020) 11:124. doi: 10.1186/s13287-020-01636-4, PMID: 32183875 PMC7079384

[8] Garcia-Arranz M Garcia-Olmo D Herreros MD Gracia-Solana J Guadalajara H Baixauli J. Autologous adipose-derived stem cells for the treatment of complex Cryptoglandular perianal fistula: A randomized clinical trial with long-term follow-up. Stem Cells Transl Med. (2020) 9:295–301. doi: 10.1002/sctm.19-0271 31886629 PMC7031651

[9] Swaroop S Vuyyuru SK Kante B Kumar P Mundhra SK Arora U. A phase I/II clinical trial of *ex-vivo* expanded human bone marrow derived allogeneic mesenchymal stromal cells in adult patients with perianal Fistulizing Crohn’s disease. Stem Cell Res Ther. (2024) 15:140. doi: 10.1186/s13287-024-03746-9 38745184 PMC11094973

[10] Barnhoorn MC Wasser MNJM Roelofs H Maljaars PWJ Molendijk I Bonsing BA. Long-term evaluation of allogeneic bone marrow-derived mesenchymal stromal cell therapy for Crohn’s disease perianal fistulas. J Crohns Colitis. (2020) 14:64–70. doi: 10.1093/ecco-jcc/jjz116, PMID: 31197361 PMC6930001

[11] Ten Bokkel Huinink S Thomassen D Steyerberg EW Pauwels RWM Casanova MJ Bouguen G. Discontinuation of anti-tumour necrosis factor therapy in patients with perianal Fistulizing Crohn’s disease: individual participant data Meta-analysis of 309 patients from 12 studies. J Crohns Colitis. (2024) 18:134–43. doi: 10.1093/ecco-jcc/jjad118, PMID: 37437094 PMC10821706

[12] Wasmann KA de Groof EJ Stellingwerf ME D’Haens GR Ponsioen CY Gecse KB. Treatment of perianal fistulas in Crohn’s disease, Seton versus anti-TNF versus surgical closure following anti-TNF [PISA]: a randomised controlled trial. J Crohns Colitis. (2020) 14:1049–56. doi: 10.1093/ecco-jcc/jjaa004, PMID: 31919501 PMC7476637

[13] Praag EMM. Short-term anti-TNF therapy with surgical closure versus anti-TNF therapy in the treatment of perianal fistulas in Crohn’s disease (PISA-II): A patient preference randomised trial. Lancet Gastroenterol Hepatol. (2022) 7:617–26. doi: 10.1016/S2468-1253(22)00088-7 35427495

[14] Newton K Dumville J Briggs M Law J Martin J Pearce L. Postoperative packing of perianal abscess cavities (PPAC2): Randomized clinical trial. Br J Surg. (2022) 109:951–7. doi: 10.1093/bjs/znac225 35929816 PMC10364677

[15] An Y Gao J Xu J Qi W Wang L Tian M. Efficacy and safety of 13 surgical techniques for the treatment of complex anal fistula, non-Crohn CAF: a systematic review and network Meta-analysis. Int J Surg Lond Engl. (2024) 110:441–52. doi: 10.1097/JS9.0000000000000776, PMID: 37737881 PMC10793738

[16] Parks AG Gordon PH Hardcastle JD. A classification of fistula-in-ano. Br J Surg. (1976) 63:1–12. doi: 10.1002/bjs.1800630102, PMID: 1267867

[17] Vogel JD Johnson EK Morris AM Paquette IM Saclarides TJ Feingold DL. Clinical practice guideline for the management of anorectal abscess, fistula-in-ano, and rectovaginal fistula. Dis Colon Rectum. (2016) 59:1117–33. doi: 10.1097/DCR.0000000000000733, PMID: 27824697

[18] Seow-Choen F Nicholls RJ. Anal fistula. Br J Surg. (1992) 79:197–205. doi: 10.1002/bjs.1800790304, PMID: 1555083

[19] Vanbeckevoort D Bielen D Vanslembrouck R Van Assche G. Magnetic resonance imaging of perianal fistulas. Magn Reson Imaging Clin N Am. (2014) 22:113–23. doi: 10.1016/j.mric.2013.07.008, PMID: 24238135

[20] Jayne DG Scholefield J Senapati A Hewitt CA. A multicenter randomized controlled trial comparing safety, efficacy, and cost-effectiveness of the Surgisis anal fistula plug versus Surgeon’s preference for Transsphincteric fistula-in-Ano: The FIAT trial. Ann Surg. (2021) 273:433–41. doi: 10.1097/SLA.0000000000003981 32516229

[21] Van der Hagen SJ Baeten CG Soeters PB van Gemert WG. Long-term outcome following mucosal advancement flap for high perianal fistulas and fistulotomy for low perianal fistulas: recurrent perianal fistulas: failure of treatment or recurrent patient disease? Int J Color Dis. (2006) 21:784–90. doi: 10.1007/s00384-005-0072-7, PMID: 16538494

[22] Dubsky PC Stift A Friedl J Teleky B Herbst F. Endorectal advancement flaps in the treatment of high anal fistula of cryptoglandular origin: full-thickness vs. mucosal-rectum flaps. Dis *Colon Rectum*. (2008) 51:852–7. doi: 10.1007/s10350-008-9242-3 18317841

[23] Jarrar A Church J. Advancement flap repair: a good option for complex anorectal fistulas. Dis *Colon Rectum*. (2011) 54:1537–41. doi: 10.1097/DCR.0b013e31822d7ddd, PMID: 22067182

[24] Abdelnaby M Emile S El-Said M Abdallah E Abdel Mawla A. Drained mucosal advancement flap versus rerouting Seton around the internal anal sphincter in treatment of high trans-Sphincteric anal fistula: a randomized trial. Int J Surg. (2019) 72:198–203. doi: 10.1016/j.ijsu.2019.11.008, PMID: 31751790

[25] Mei Z Wang Q Zhang Y Liu P Ge M Du P. Risk factors for recurrence after anal fistula surgery: a Meta-analysis. Int J Surg. (2019) 69:153–64. doi: 10.1016/j.ijsu.2019.08.003, PMID: 31400504

